# Prevalence of Artificial Intelligence-Generated Text in Neurosurgical Publications: Implications for Academic Integrity and Ethical Authorship

**DOI:** 10.7759/cureus.79086

**Published:** 2025-02-16

**Authors:** Daniel M Schneider, Akash Mishra, Jacob Gluski, Harshal Shah, Max Ward, Ethan D Brown, Daniel M Sciubba, Sheng-Fu L Lo

**Affiliations:** 1 Department of Neurosurgery, Donald and Barbara Zucker School of Medicine at Hofstra/Northwell, Manhasset, USA

**Keywords:** academic integrity, academic neurosurgery, academic publishing, artificial intelligence, ethics

## Abstract

Introduction: With the rapid proliferation of artificial intelligence (AI) tools, important questions about their applicability to manuscript preparation have been raised. This study explores the methodological challenges of detecting AI-generated content in neurosurgical publications, using existing detection tools to highlight both the presence of AI content and the fundamental limitations of current detection approaches.

Methods: We analyzed 100 randomly selected manuscripts published between 2023 and 2024 in high-impact neurosurgery journals using a two-tiered approach to identify potential AI-generated text. The text was classified as AI-generated if both a robustly optimized bidirectional encoder representations from transformers pretraining approach (RoBERTa)-based AI classification tool yielded a positive classification and the text’s perplexity score was less than 100. Chi-square tests were conducted to assess differences in the prevalence of AI-generated text across various manuscript sections, topics, and types. In an effort to eliminate bias introduced by the more structured nature of abstracts, a subgroup analysis was conducted that excluded abstracts as well.

Results: Approximately one in five (20%) manuscripts contained sections flagged as AI-generated. Abstracts and methods sections were disproportionately identified. After excluding abstracts, the association between section type and AI-generated content was no longer statistically significant.

Conclusion: Our findings highlight both the increasing integration of AI in manuscript preparation and a critical challenge in academic publishing as AI language models become increasingly sophisticated and traditional detection methods become less reliable. This suggests the need to shift focus from detection to transparency, emphasizing the development of clear disclosure policies and ethical guidelines for AI use in academic writing.

## Introduction

The integration of artificial intelligence (AI) tools in academic writing presents a fundamental challenge to traditional concepts of authorship and academic integrity. While these tools offer opportunities to enhance research productivity and writing clarity, their rapid evolution - particularly with advanced language models - has created a complex landscape where the distinction between human and AI-generated text becomes increasingly ambiguous [1]. This ambiguity raises critical questions about how academic publishing should adapt to a reality where reliable detection of AI-generated content may be fundamentally impossible.

Previous approaches to maintaining academic integrity, such as plagiarism detection, relied on identifying copied text through pattern matching [2]. However, AI-generated content presents a fundamentally different challenge - the text is often original, contextually appropriate, and increasingly indistinguishable from human writing [3]. This technological advancement necessitates a reimagining of how academic publishing approaches AI integration.

This evolution coincides with the significant increase in academic publications each year [4-5], particularly in academic medicine, where peer-reviewed publications remain essential for professional advancement [6-7]. While AI-powered tools offer authors means to streamline writing and ensure coherent texts [1-8], they also raise complex questions about authenticity and verification that traditional quality control measures may be ill-equipped to address.

In fields like neurosurgery, where precision and thorough documentation are critical [6], AI tools are becoming valuable resources for reducing the workload associated with clinical and academic writing [9]. Journals must now grapple with content that may be partially or wholly AI-generated [10-12]. The increasing quality and accessibility of these tools create an urgent need for new frameworks to ensure scientific integrity [13-14].

While many journals now require disclosure of AI use in manuscript preparation [8,15-16], the effectiveness of these policies relies heavily on author compliance. The challenge is compounded by the rapid advancement of AI technology, which makes reliable detection increasingly difficult. This creates a fundamental tension between leveraging AI's benefits while maintaining the integrity and transparency of academic publishing.

This study examines the methodological challenges of detecting AI-generated content in neurosurgical literature through a two-fold approach: first, by applying current detection methods to quantify potentially AI-generated content, and second, by using these results to illustrate the fundamental limitations of detection-based approaches. Rather than claiming definitive identification of AI-generated text, we aim to demonstrate why the academic community should shift focus from detection to transparency and ethical guidelines.

## Materials and methods

Data collection

PubMed was queried to identify manuscripts for AI-generated text detection. The search was limited to articles appearing in high-impact journals with a neurosurgical or spine focus. These journals were *Journal of Neurosurgery*, *Neurosurgery*, *Operative Neurosurgery*, *Spine*, and *World Neurosurgery*. Screening criteria included the English language and a publication date between January 2023 and December 2024. Our search was limited to specific article types such as review articles, meta-analyses, and case reports. These article types were chosen because they typically contain longer narrative sections and may be more conducive to the identification of AI-generated text. Articles with alternative formatting were also excluded. A total of 774 articles met all inclusion criteria. We randomly selected 100 of these articles by assigning each manuscript a number between 1 and 774 using MATLAB R2024a (MathWorks Inc., Natick, USA) and selecting the first 100 manuscripts.

Manuscript text extraction and chunking

The text was extracted from manuscript PDFs and subsequently segmented based on the following manuscript sections: introduction, methods, results, discussion, and conclusion. To comply with the input size limitations of the AI detection models used, each section was chunked into 200-word segments.

Methodological approach rationale

Our approach to AI detection intentionally utilizes methods that were validated on earlier AI models to illustrate a key challenge in the field. While these methods demonstrated high accuracy with previous-generation language models, their effectiveness against current models remains largely unknown. This limitation serves as a central point in our discussion of the evolving challenges in AI detection.

AI-generated text detection model selection

Two commonly employed methods in the domain of AI-generated text are 1) robustly optimized bidirectional encoder representations from transformers pretraining approach (RoBERTa)-based analysis and 2) perplexity analysis. RoBERTa is a transformer-based language model that has been fine-tuned for text classification tasks such as distinguishing AI-generated content. RoBERTa detects subtle statistical irregularities in word patterns that often emerge in machine-generated text. According to Liu et al. (2019), a fine-tuned RoBERTa classifier achieves a sensitivity of 85%, a specificity of 90%, and a false positive rate of 10% [17].

Perplexity analysis evaluates how well a language model predicts the next word in a sequence, with low perplexity indicating higher predictability. Machine-generated text tends to have lower perplexity because the language model generating it is highly confident about the next word based on the sequence. Human-written text, on the other hand, exhibits more variability and thus higher perplexity. While there is no standard perplexity threshold to indicate AI-generated text, lower values tend to correlate well with AI-generated text. Ippolito et al. (2020) observed that perplexity-based detectors can achieve a sensitivity of approximately 80%, a specificity of 90%, and a false positive rate of 10% [18], though more recent investigations have suggested lower values [19].

Using RoBERTa-based analysis and perplexity analysis together aims to reduce false positives at the cost of some sensitivity. Based on the available literature on the individual approaches, the combined approach is estimated to have a sensitivity of 68%, a specificity of 99%, and a false positive rate of 1%.

A section was considered to be AI-generated if it met both of the following criteria: (1) the RoBERTa model classified the section to be likely AI-generated and (2) the perplexity score was less than 100, based on the 25th percentile threshold of all manuscripts.

Statistical analysis

In the initial analysis, all manuscript sections were considered; a subsequent analysis was run excluding abstract sections. A chi-square test was used to assess whether there were significant associations between manuscript sections and the likelihood of being flagged as AI-generated content. Descriptive statistics for perplexity scores were calculated, and the proportion of sections that were flagged as being AI-generated was reported. All statistical analyses were performed in MATLAB R2023a (MathWorks Inc., Natick, USA). Text was extracted from the PDF using the PyPDF2 and pdfplumber libraries. The software environment was Windows 11 running Python 3.0 (Python Software Foundation, Wilmington, USA) with the following additional libraries being employed: Pandas, Matplotlib, SciPy, Torch, and Sklearn.

## Results

**Figure 1:**
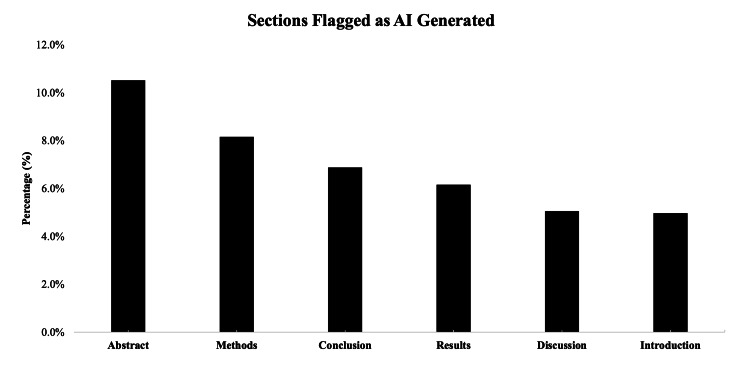
Manuscript sections flagged as AI-generated A bar chart showing the section categories (abstract, methods, conclusions, results, discussion, and introduction) on the X-axis, and the percentage that were flagged by our analysis as being AI-generated is represented on the Y-axis. AI: artificial intelligence

The inclusion criteria returned 774 articles, of which 100 articles were randomly selected for further analysis. Each subsection of each article (abstract, introduction, methods, results, and discussion/conclusion) was then divided into 200-word segments for AI-generated content detection. A total of 2452 article sections were assessed, with the largest number of segments coming from results sections (n=860; 35.1%) followed by discussion sections (n=553; 22.6%). The least number of segments were from abstract sections (n=171; 7.0%). The algorithm flagged a total of 41 sections (1.7%) as being likely AI-generated. Flagged sections had a mean perplexity score of 44.5±32.2 (range: 3.7-99.0). A chi-square test revealed a significant association between article section type and being flagged as AI-generated (X^2^(4)=52.4, p<0.001). The article sections most likely to be identified as containing AI-generated content were abstracts (n=14; 8.2%) followed by methods (n=9; 2.1%) (Table 1; Figure 1).

**Table 1:** Distribution of analyzed and flagged sections by manuscript section type This table summarizes the number of sections analyzed, the number flagged as AI-generated, and the corresponding percentage for each section type across 100 neurosurgical manuscripts. Abstracts had the highest percentage of flagged content, with 8.19% of sections identified as AI-generated, compared to 1.67% overall. This highlights the structured nature of abstracts, which may increase the likelihood of AI detection. AI: artificial intelligence

Section	Analyzed	Flagged	Percentage
Results	860	6	0.70
Discussion	553	9	1.63
Methods	429	9	2.1
Introduction	221	1	0.45
Conclusion	218	2	0.92
Abstract	171	14	8.19
Total	2452	41	1.67

**Figure 2:**
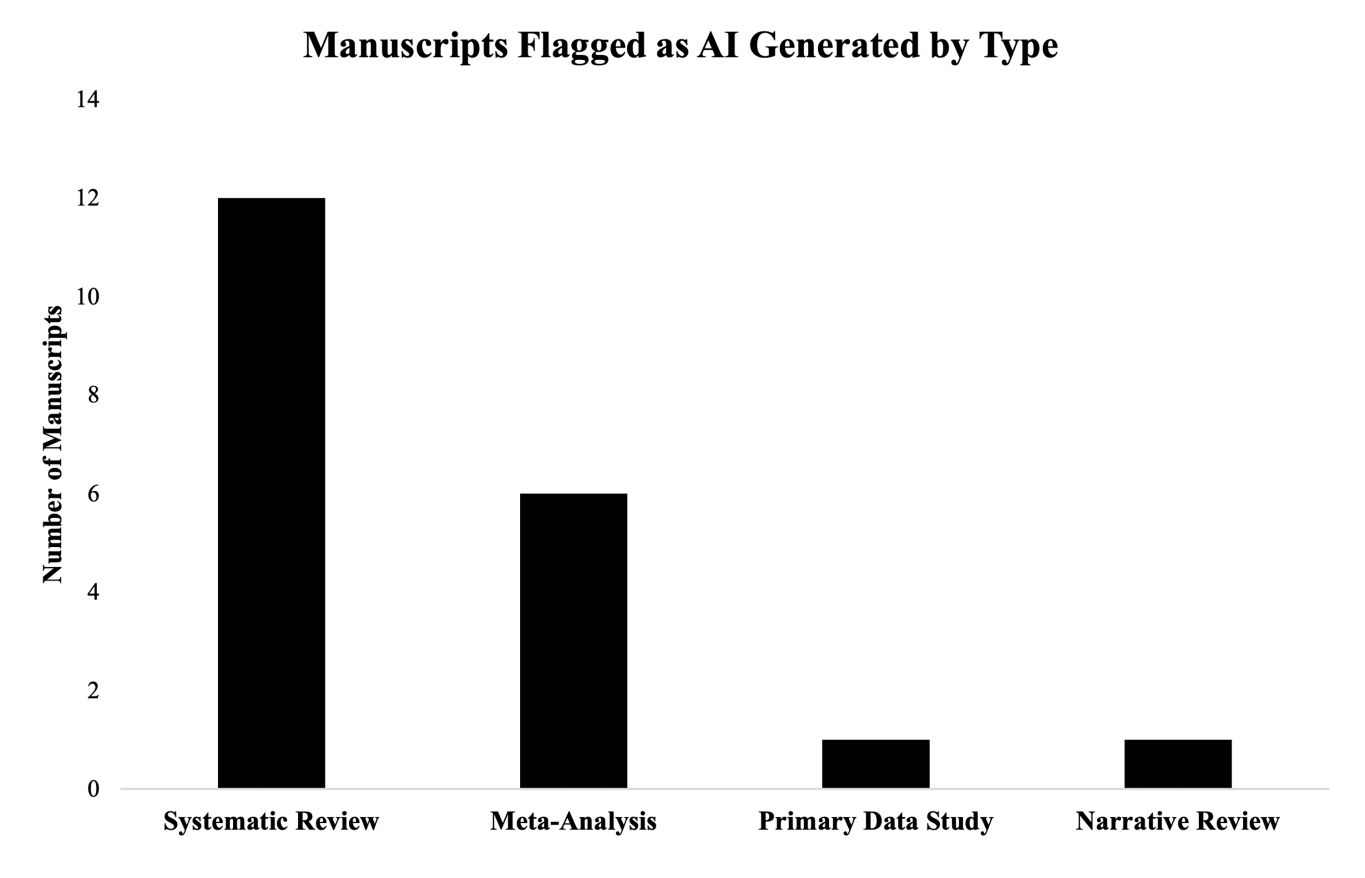
Flagged manuscripts by type A bar chart showing the frequency at which different article types were flagged by our analysis as having AI-generated content. The X-axis represents the type of article flagged (systematic reviews, meta-analyses, original scientific articles, and narrative reviews) while the Y-axis represents the number of articles. AI: artificial intelligence

**Figure 3:**
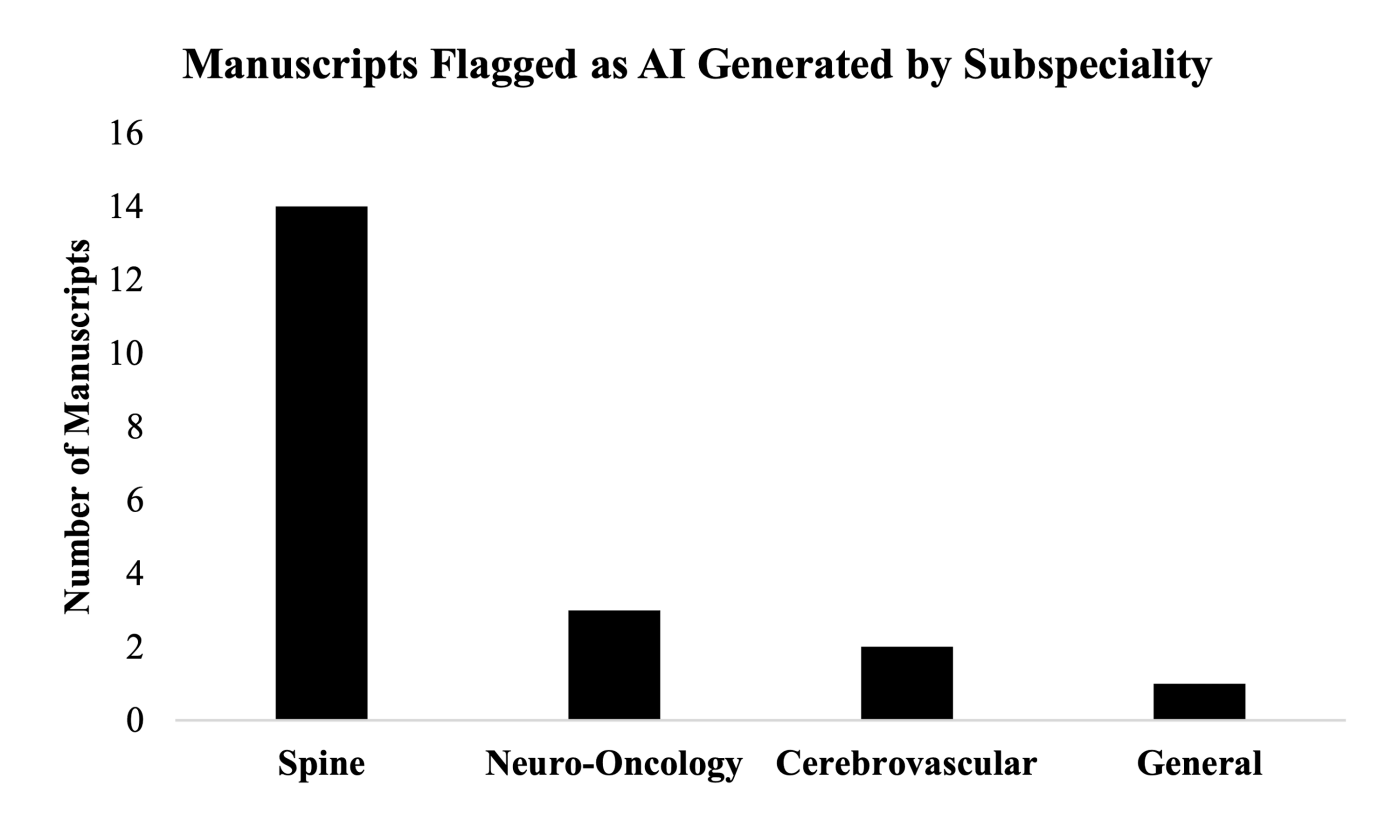
Flagged manuscripts by subspecialty A bar chart showing the frequency at which different subspecialty topics (spine, neuro-oncology, cerebrovascular, and general) were flagged as having AI-generated content wherein the X-axis represents the subspecialty while the Y-axis represents the number of articles. AI: artificial intelligence

A total of 20 manuscripts (out of 100; 20%) were found to contain a section that contained AI-generated content (Table 2). The manuscript type that was most commonly flagged to contain AI-generated content was systematic reviews (n=12; 60.0%) followed by meta-analyses (n=6; 30.0%) (Figure 2). When manuscripts were assessed for subject matter, it was found that most articles that were flagged to be AI-generated came from spine-focused publications (n=14; 70.0%) followed by neuro-oncology (n=3; 15.0%) (Figure 3). Due to the structured nature of abstracts potentially leading the AI detection tools to result in false positives, a separate subgroup analysis was performed excluding abstracts. In this analysis, 17 manuscripts (17%) were found to contain AI-generated content.

**Table 2:** Distribution of article types and subspecialties among flagged manuscripts The table shows a breakdown of the individual manuscripts flagged as having AI-generated content, specifying the article type and subspecialty. AI: artificial intelligence

Manuscript	Article type	Subspecialty
5	Primary data study	Neuro-oncology
8	Meta-analysis	Spine
11	Systematic review	Spine
14	Systematic review	Neuro-oncology
17	Meta-analysis	Spine
26	Systematic review	Spine
34	Systematic review	Spine
40	Meta-analysis	Spine
47	Systematic review	Spine
49	Narrative review	Spine
60	Systematic review	Cerebrovascular
65	Systematic review	Spine
66	Systematic review	Spine
71	Systematic review	General
72	Meta-analysis	Neuro-oncology
84	Systematic review	Spine
85	Meta-analysis	Cerebrovascular
87	Meta-analysis	Spine
89	Systematic review	Spine
97	Systematic review	Spine

## Discussion

Our study reveals two critical findings about AI in academic publishing: first, that AI-generated content is likely present in peer-reviewed literature at a detectable level, and second, perhaps more importantly, that reliable detection of such content may be fundamentally unreliable as AI technology rapidly evolves. The detection methods employed in this study, while state-of-the-art when developed for earlier models [3,9,20] face significant limitations when applied to content potentially generated by more recent AI models. This limitation highlights a crucial challenge facing academic publishing - the growing impossibility of reliable AI detection - without undermining our study's value [21-23].

As language models continue to advance, they increasingly excel at producing the structured prose found in scientific manuscripts [24]. The ability to compose structured prose has particular utility for abstracts, methods, and conclusions - sections where our analysis flagged the highest proportion of potentially AI-generated content. However, the very structured nature of scientific writing that makes it amenable to AI generation also complicates detection efforts, as both human and AI authors strive to meet the same stylistic and organizational conventions [11].

The application of AI tools in manuscript generation extends beyond initial drafting [21]. Tools such as AI-based grammar editors, style editors, and plagiarism detectors are now routinely used by authors to refine their manuscripts prior to submission [2]. While these technologies blur the line between AI assistance and human authorship, they also could have a positive impact.

Implications for academic publishing

Given these challenges, academic publishing may need to evolve beyond detection-based approaches. Many journals already have official policies requiring the disclosure of AI use, but no standards exist on how to convey these disclosures to readers. The need for further adoption and adherence to rigorous guidelines for AI tools within scientific writing is driven by two main factors: 1) Generative AI frequently suffers from hallucinations, which could accidentally introduce falsehoods into a manuscript [15]. The detection of these hallucinations is not always easy and would require a careful critical review of AI-generated content; 2) The use of generative AI raises broader issues of authorship and originality that challenge traditional concepts of academic writing.

Future directions

Based on our findings, we propose several key considerations for the academic publishing community: 1) The development of standardized disclosure frameworks should specify not only the use of AI tools but also detail the degree and nature of AI assistance; 2) The creation of validation protocols for AI-generated content should focus on accuracy and scientific integrity rather than detection; 3) Guidelines for appropriate AI integration should maximize benefits while maintaining academic rigor; and 4) The evolution of peer review processes should account for potential AI involvement.

Limitations

This study has several important limitations. First, as large language models (LLMs) such as Generative Pre-trained Transformer (GPT)-4 continue to advance, the task of distinguishing AI-generated text from human-authored content becomes increasingly challenging. The estimated sensitivity of 68% in our study was derived from models detecting text generated by earlier iterations of LLMs, such as GPT-2 and GPT-3. As newer AI models continue to improve in their ability to generate human-like text, the sensitivity of our detection methods may be further diminished. This could result in an underestimation of the true prevalence of AI-generated text in neurosurgical manuscripts [23,25]. Similarly, the possible inclusion of studies submitted before the public release of GPT-3.5 in November 2022 may have contributed to the underestimation of AI-generated text.

Additionally, the perplexity threshold we employed, while effective for our sample, may not be fully generalizable to other datasets or text domains. This threshold was optimized for the specific corpus used in our study and may require adjustment when applied to texts outside this context. Importantly, the AI-detection models we used were not specifically trained in neurosurgical literature, which contains highly specialized jargon, terminology, and structural conventions. This domain-specific language may reduce the accuracy of AI detection when applied to texts in the neurosurgical field, where the complexity and technical specificity of the content could confound existing models [11,25].

Finally, for methodological standardization, we divided the texts into 200-word chunks for analysis. While this approach ensured compatibility across models, the arbitrary division of text may disrupt the syntactic flow and coherence of the content, potentially impairing the model’s ability to accurately detect AI-generated material [11]. Future studies should explore content-aware segmentation techniques to preserve syntactic and contextual integrity, which may enhance detection accuracy and improve the overall classification performance.

## Conclusions

This study highlights the complex challenges academic publishing faces in the era of advanced AI language models. While our analysis detected potential AI-generated content in approximately one in five neurosurgical papers, these findings primarily serve to illustrate two fundamental points: that AI-generated content is likely present in peer-reviewed literature at a detectable level and that there exists a growing difficulty of reliable AI detection in academic publishing. As language models continue to evolve, traditional detection methods become increasingly inadequate for ensuring manuscript authenticity.

This technological reality suggests that the academic community must shift focus from detection to transparency. Rather than relying on increasingly unreliable detection tools, the field needs to develop robust frameworks for ethical AI use in scholarly work. These frameworks should address not only disclosure requirements but also establish guidelines for appropriate AI integration, validation protocols for AI-assisted content, and methods for maintaining scientific integrity. While AI tools offer potential benefits for improving research communication and productivity, their integration into academic publishing must be guided by clear principles and practices that prioritize transparency over detection.
